# Penguins significantly increased phosphine formation and phosphorus contribution in maritime Antarctic soils

**DOI:** 10.1038/srep07055

**Published:** 2014-11-14

**Authors:** Renbin Zhu, Qing Wang, Wei Ding, Can Wang, Lijun Hou, Dawei Ma

**Affiliations:** 1 Institute of Polar Environment, School of Earth and Space Sciences, University of Science and Technology of China, Hefei City, Anhui Province 230026, P. R China; 2 State Key Laboratory of Estuarine and Coastal Research, East China Normal University, Shanghai 200062, P. R China

## Abstract

Most studies on phosphorus cycle in the natural environment focused on phosphates, with limited data available for the reduced phosphine (PH_3_). In this paper, matrix-bound phosphine (MBP), gaseous phosphine fluxes and phosphorus fractions in the soils were investigated from a penguin colony, a seal colony and the adjacent animal-lacking tundra and background sites. The MBP levels (mean 200.3 ng kg^−1^) in penguin colony soils were much higher than those in seal colony soils, animal-lacking tundra soils and the background soils. Field PH_3_ flux observation and laboratory incubation experiments confirmed that penguin colony soils produced much higher PH_3_ emissions than seal colony soils and animal-lacking tundra soils. Overall high MBP levels and PH_3_ emissions were modulated by soil biogeochemical processes associated with penguin activities: sufficient supply of the nutrients phosphorus, nitrogen, and organic carbon from penguin guano, high soil bacterial abundance and phosphatase activity. It was proposed that organic or inorganic phosphorus compounds from penguin guano or seal excreta could be reduced to PH_3_ in the Antarctic soils through the bacterial activity. Our results indicated that penguin activity significantly increased soil phosphine formation and phosphorus contribution, thus played an important role in phosphorus cycle in terrestrial ecosystems of maritime Antarctica.

Phosphorus (P) is an essential nutrient frequently limiting primary production for terrestrial and aquatic ecosystems^1^. Most studies on phosphorus cycle in the natural environment focused on +5 valence phosphates in aqueous and solid forms, while limited research on volatile phosphorus compounds has been conducted^1^,^2^. Phosphine (PH_3_) is a kind of reactive and reduced phosphorus component, and has been recognized as a gaseous carrier of phosphorus in global biogeochemical cycles in two different forms: free gaseous phosphine (FGP) and matrix-bound phosphine (MBP)^2^,^3^,^4^,^5^,^6^,^7^. MBP, defined as phosphine released from condensed environmental samples, can be hydrolyzed in biological aquatic media to form FGP by acid or alkaline digestion^2^,^8^,^9^. At present, considerable studies have demonstrated the efforts of phosphine emissions from sewage treatment plants, animal slurry, landfills, communal waste, sea sediments, lake sediments and paddy fields^3^,^4^,^5^,^6^,^10^,^11^,^12^,^13^,^14^,^15^,^16^,^17^,^18^,^19^. Several reports claim that phosphine can be produced by biochemical processes, such as the bacterial reduction of phosphates or natural organophosphorus compounds^16^,^17^,^20^,^21^,^22^,^23^. Phosphine emissions and its forming biochemical processes support a gaseous link to the phosphorus biogeochemical cycle in the global environment^2^.

In coastal Antarctica, some ice-free coastal zones are colonized by a large number of marine animals like penguins and seals. Marine animal colonies, tundra vegetation (mosses, lichens and algae) and their interactions form a special tundra ecosystem. The soils within penguin colonies are described as ornithogenic due to the presence of organic materials including guano, feathers and eggshells^24^. Penguin guano and ornithogenic soils are the most important phosphorus-rich matrixes in Antarctic ecosystems^24^,^25^,^26^. Recently, high MBP concentrations and phosphine emission rates have been preliminarily explored from penguin and seal colony soils^6^,^27^. Nevertheless, such data are available only from very limited site level observations, and few studies have been conducted to investigate the impact of marine animal activities on soil MBP distribution patterns, phosphine emission rates and phosphorus bio-transportation in maritime Antarctica. The phosphine from marine animal colonies could be a small but significant carrier of the nutrient phosphorus in maritime Antarctic tundra ecosystem^6^. It is very necessary to explore whether matrix-bound phosphine (MBP) and gaseous phosphine (FGP) under the disturbance of marine animal activities contribute to a certain extent to the geochemical cycle of phosphorus in maritime Antarctica.

In this paper, soil MBP and phosphorus levels were investigated in a penguin colony, a seal colony and their adjacent animal-lacking tundra on Ardley Island and Fildes Peninsula in maritime Antarctica. The in-situ phosphine emission rates and in-vivo phosphine production rates in the soils were measured from their colonies and animal-lacking tundra sites based upon field observations and laboratory incubation experiments. The study area and all the sampling sites were illustrated in Fig. 1. The objectives of this study were (1) to explore the effects of penguin and seal activities on soil MBP levels, and gaseous phosphine production and emission in maritime Antarctica; (2) to investigate the phosphorus contribution of penguins and seals from sea to land; and (3) to discuss the potential formation mechanisms of phosphine in maritime Antarctic soils.

## Results

### Contribution of penguins to soil phosphorus

Total phosphorus (TP), organic phosphorus (OP) and inorganic phosphorus (IP) concentrations in penguin colony soils and the adjacent penguin-lacking tundra soils on Ardley Island were illustrated in Fig. 2. The TP, OP and IP concentrations in the soils showed a large spatial variability due to penguin activities. Extremely high phosphorus levels (mean 42.9 mg g^−1^) occurred in the active penguin colony soils in the east of Ardley Island, and the OP contents (mean 28.7 mg g^−1^) were almost twice higher than IP contents (14.3 mg g^−1^). Generally the contents of phosphorus fractions (TP, OP and IP) in penguin colony soils were significantly higher than those in the adjacent penguin-lacking tundra soils (<20 mg g^−1^), seal colony soils (<3 mg g^−1^) and the local background soils (<2 mg g^−1^) (Fig. 3b, c, d). The contribution rates of penguins to soil phosphorus fractions (TP, OP and IP) amounted to 86%–98%, evidently higher than those of seals (27%–77%) in maritime Antarctica (Table 1).

Higher phosphatase activity (PA) occurred in penguin colony soils (mean 277.4 mg kg^−1^ h^−1^) than in the adjacent tundra soils (mean 198.4 mg kg^−1^ h^−1^) (Fig. 2e). Overall PA in penguin colony soils and the adjacent tundra soils was much higher than those in seal colony soils (mean 55.5 mg kg^−1^ h^−1^) and background soils (38.4 mg kg^−1^ h^−1^). The invertase activity (IA) in the soils also showed similar distribution patterns (Fig. 3e, f). The weak PA and IA in seal colony soils might be due to low levels of soil TP, OP and IP. Our results indicated that penguin activity might have a stronger effect on tundra soil P biogeochemical properties than seal activity in our study area.

In addition, total nitrogen, total carbon and total sulfur contents in penguin colony soils were significantly higher than those in the adjacent penguin-lacking tundra soils, seal colony soils and the local background soils (Supplementary Table S1). The mean NH_4_^+^–N and NO_3_^−^–N concentrations in penguin colony soils were one to two orders of magnitude higher than those in seal colony soils and local background soils. Penguin activity decreased soil pH and carbon/nitrogen ratio, but increased soil moisture. Furthermore, phosphorus fractions, and total nitrogen, total organic carbon and total sulfur contents, PA and IA showed a significant positive correlation with each other (Supplementary Table S2).

### MBP levels in penguin and seal colony and animal-lacking tundra soils

Matrix-bound phosphine (MBP) existed in all the Antarctic soil samples. Much higher MBP levels generally occurred in penguin colony soils (mean 200.3 ng kg^−1^) than in the adjacent penguin-lacking tundra soils (mean 137.4 ng kg^−1^) on Ardley Island (Fig. 2a), whereas relatively lower MBP levels concentrated in seal colony soils (mean 101.6 ng kg^−1^) and the background soils (mean 75.5 ng kg^−1^) (Fig. 3a). The MBP concentrations in the soils showed a significant positive correlation with TP (P<0.0001), OP (P<0.0001), IP (P<0.0001), TN (P<0.0001), TC (P<0.0001) and TS (P<0.0001) when all the data were combined (Supplementary Fig. S1). Therefore penguin activity and the nutrients from penguin guano significantly increased soil MBP levels. Compared to the local background soils, the contribution rate of penguins to soil MBP was 62.3%, much higher than that of seals (25.7%) in their colonies (Table 1).

### In-situ phosphine fluxes from penguin and seal colony and animal-lacking tundra soils

Phosphine was emitted in situ under the Antarctic environment conditions from penguin and seal colony soils and their adjacent tundra soils at a rate in the order of 20–160 ng m^−2^ h^−1^. It was noted that PH_3 _fluxes from penguin colony soils were more than two times larger than those from seal colony soils and their adjacent animal-lacking tundra soils. These emission rates were significantly higher than those (<10 ng m^−2^ h^−1^) from the local background soils (Fig. 4a). Penguins increased tundra soil PH_3_ fluxes by twenty times compared to the background soils, more than two times higher than seals (Table 2). Combined with the exploration of high phosphine emission rates from penguin colony soils, we propose that the emissions from penguin colonies might be the predominant sources for atmospheric phosphine in maritime Antarctica. The final fate of atmospheric phosphine will be oxidation into water-soluble phosphate, which might enter the adjacent penguin-lacking tundra ecosystem, thus increase tundra soil TP, OP and IP contents in maritime Antarctica (Fig. 3).

### In-vivo phosphine production rates in penguin and seal colony and animal-lacking soils

As illustrated in Fig. 4b, penguin colony soils showed much higher phosphine production rate than seal colony soils and their adjacent animal-lacking tundra soils. Overall phosphine production rates from penguin and seal colony soils and their adjacent tundra soils were one order of magnitude higher than those from the background tundra soils. Penguins increased tundra soil PH_3_ production rates by twenty-five times compared to the background soils, almost three times higher than seals (Table 2). MBP formation and gaseous phosphine production might have positive distributing effects on the dissemination of phosphorus in maritime Antarctica. The redistribution of phosphorus via phosphine from animal colonies could additionally fertilize adjacent tundra areas.

### Potential formation mechanism of phosphine in Antarctic soils

The bacterial abundance in penguin and seal colony soils and their adjacent tundra soils ranged from 2.5 × 10^10^ to 1.23 × 10^12^ gene copies g^−1^ soil (Fig. 5a). The gene copies in penguin colony soils were one to two orders of magnitude higher than those in seal colony soils, their adjacent tundra soils and the local background soils. There is a significant positive correlation between MBP concentrations and the bacterial abundance, invertase and phosphatase activity (Fig. 5b, c, d). Our results confirm that the production of PH_3_ is associated with microbial activity, and all kinds of soil organic or inorganic phosphorus compounds from penguin guano or seal excreta can be reduced to PH_3_ through the bacterial activity under the Antarctic environmental conditions.

## Discussion

To our knowledge, the reported soil matrix-bound phosphine levels and PH_3_ fluxes represent the first detailed comparative analyses of potential phosphorus exchange from marine animal colonies and the adjacent animal-lacking tundra in maritime Antarctica. Our results clearly showed that penguin colony soils had greater potential for MBP formation and PH_3_ emissions than seal colony and animal-lacking tundra soils. At present, the available studies on soil MBP and phosphorus levels for maritime Antarctica have concentrated more upon the limited tundra sites^6^,^27^. Here we studied the spatial distribution patterns of soil MBP and phosphorus levels and their affecting factors from marine animal colony-tundra ecosystems with better spatial coverage than had ever been done before. Therefore this study contributes to the knowledge about phosphorus cycle in maritime Antarctica.

The MBP concentrations are in the range of 63.2–433.6 ng kg^−1^ dw in penguin and seal colony soils and the adjacent animal-lacking tundra soils. Our data are comparable to those in freshwater sediments of Hamburg harbor^15^ and Elster River in Germany^2^ and Taihu in China^5^, and in marine surface sediments of German Bight^28^ and Chinese coastal areas, and even of seriously polluted areas in Chinese Jiaozhou Bay^29^ (Supplementary Table S3). Furthermore, MBP concentrations in this study are higher than those in the soils of industrial and rural areas in Germany^4^, paddy soils in China^30^, virgin tropical forest soils of Mahé in Seychelles^2^, and estuarine intertidal sediments of the Yangtze River^10^. Even low concentrations of soil MBP could be important, assuming they indicate a stationary state concentration of phosphine between production and consumption under natural conditions^2^,^6^. Therefore, in this study high MBP concentrations imply that MBP cannot be ignored for the phosphorus cycle in penguin colony soils.

In maritime Antarctica, penguins or seals provide considerable external P and N inputs for their colonies and adjacent tundra soils through the direct input of their guano/excreta and atmospheric transport^24^,^25^,^26^. Generally the productivity of Antarctic ecosystems is limited strongly due to extremely low N and P levels^24^,^26^,^31^. Significantly elevated TP, IP and OP concentrations occurred in penguin colony soils and the adjacent tundra soils as compared with local background soils (Fig. 3). A strong positive correlation between MBP concentrations and soil TP, IP, OP and TN contents further confirms that marine animal-derived P and N input is the predominant factor controlling the spatial variability in soil MBP concentrations (Fig. S1). Previous studies also showed that there existed a significant positive correlation between MBP and TP, OP or IP concentrations in estuary and marine sediments^10^,^11^,^18^,^19^, and adding phosphate enhanced the release of soil PH_3_ in some laboratory simulation experiments^4^,^17^,^23^. In this study, PH_3_ emission rates from penguin colony soils were much higher than those from seal colony soils, the adjacent animal-lacking tundra soils and the background soils (Fig. 4). As reported in the literatures, the emission rates from penguin and seal colony soils and animal-lacking tundra soils were significantly higher than those (0.42–6.52 ng m^−2^ h^−1^) from some saltmarshes, lake sediments and paddy fields^3^,^5^,^14^. The fluxes from penguin colony soils were comparable or even greater than our previous results from the emperor penguin colony and seal colony^6^. Penguin colony soils had larger and more sustaining PH_3_ production rates than animal-lacking soils based upon our incubation experiments, indicating that the PH_3_ fluxes from penguin colonies were probably sustained by the continuous P input of penguin guano. Generally penguin and seal colonies and their active areas are devoid of vegetation due to toxic overmanuring and trampling. Higher PH_3_ emissions from penguin colonies were supported by the absence of plant P uptake, together increasing availability of P for PH_3_ production (Fig. 2). Therefore penguin activity and the deposition of their guano significantly enhanced soil MBP formation and PH_3_ emissions from the local tundra soils, and furthermore altered tundra ecosystem P cycles in maritime Antarctica.

The MBP production is generally assumed to relate to the microbial reduction of phosphate and the decomposition of organic phosphorus compounds under anaerobic conditions^2^,^16^,^17^. The anaerobic incubation experiments based on different inocula gave the evidence for the existence of microbially mediated production of phosphine^20^,^21^,^22^,^23^. In this study, MBP concentrations showed significant positive correlations with the bacterial abundance (Fig. 5b), indicating that MBP formation in tundra soils was associated with bacterially mediated processes^21^,^22^. The invertase is an important enzyme for regulating carbon cycle by catalyzing the hydrolysis of sucrose, the phosphatase plays an important role in the biological liberation of phosphorus in soil systems, and directly affects the decomposition and transformation of soil organic phosphorus and its bioavailability^32^,^33^. The close relations between MBP levels and invertase and phosphatase activities further indicated the bacterially mediated formation of MBP in Antarctic soils (Fig. 5c, d). Additionally, TC, TN and TS in the soils are important sources of carbon, nitrogen and energy for the microbial metabolism in Antarctic terrestrial ecosystems, and their significant positive correlations with MBP concentrations might reflect active microbial processes correlated to PH_3_ production^11^,^12^,^13^,^14^,^21^,^22^. Phosphine production in sediments or culture media could be enhanced with the addition of easily decomposable organic matter^4^,^17^. The deposition of penguin guano or seal excreta into tundra soils probably contributed to the input of organic carbon, nitrogen and sulfur, and the increase in bacterial activity, thus increased MBP production and gaseous PH_3_ emissions in maritime Antarctica.

Generally MBP is a reduced, unstable phosphorus compound, and easily converted to other phosphorus compounds. It can be considered as a sub-stationary state concentration of PH_3_ between production and consumption, and only a small residue of the PH_3_ turnover could be observed as MBP in the soils^2^. Under such circumstances, a slow migration process of PH_3_ is possible in the interstitial gas of tundra soils, thus influence the phosphorus balance in the Antarctic tundra ecosystem^2^,^6^. Although the quantity of PH_3_ is insignificant compared to TP in this study, its influence on the transfer and nutrition preservation of P could be important over a long period. Furthermore, the favorable conditions for high MBP production are created by physical and chemical processes related to penguin activity: sufficient supply of P, N and organic carbon from penguin guano, strong bacterial activity, penguin tramp and anaerobic environment, and can increase PH_3_ fluxes from soils to the atmosphere^6^,^34^. Therefore MBP is an important gaseous link in the P biogeochemical cycles in ornithogenic tundra ecosystems of Antarctica. The ornithogenic soils are particularly rich in phosphorus. It is estimated that penguins alone can carry 1.5 × 10^4^–2.0 × 10^4^ tons of P annually to the land via discharging guano^27^. The coupled production and oxidation process of PH_3_ in ornithogenic soils might act as an alternative shunt associated with the P cycling, and provide a small amount of phosphate for the P-limiting tundra ecosystems in Antarctica^6^,^25^,^27^. The study of MBP levels and PH_3_ fluxes in ornithogenic soils/sediments will improve our understanding of P species and P cycling in Antarctic tundra ecosystems.

## Methods

### Study area

**Figure 1:**
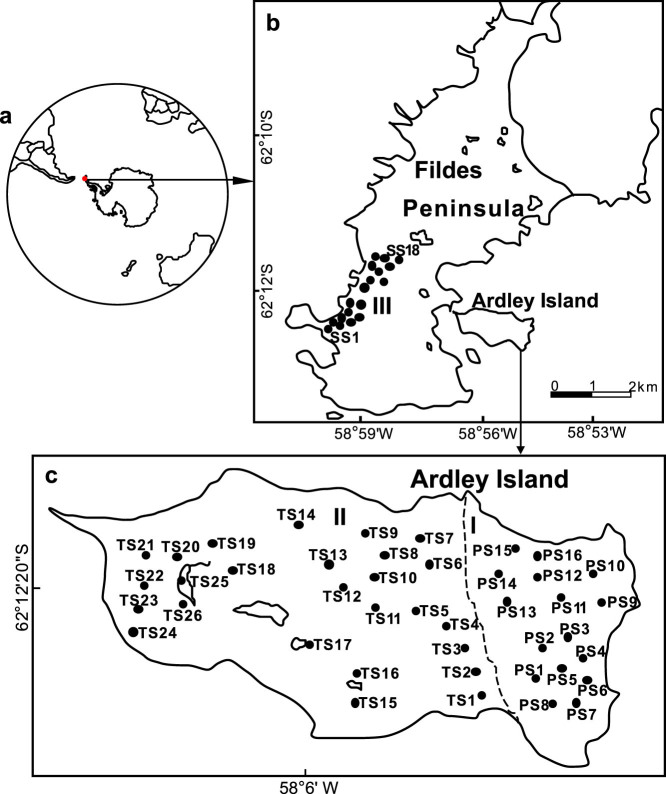
Study area and the sampling sites: (a) The red dot indicates location of the investigation area in maritime Antarctica. (b) Location of the sampling sites: Ardley Island and Fildes Peninsula, showing the tundra soil sampling sites. I: The sixteen sites PS1-PS16 at active penguin colony, and II: The twenty-six sites TS1-TS26 at the adjacent tundra lacking visible penguin and other animal activities on Ardley Island; III: The eighteen sites SS1-SS18 at seal colony, and the adjacent tundra lacking visible seal and other animal activities along the western coast on Fildes Peninsula; IV: The four background sites BS1-BS4 in the preglacial area of Nelson Island and the upland tundra faraway from animal colonies, and these sites are not shown in the figure. The boundary of areas I and II was determined according to our field investigation in Antarctica^34^. *The map was drawn using Microsoft Excel 2010 and then converted to eps format using Microsoft Office Visio 2007.

**Figure 2:**
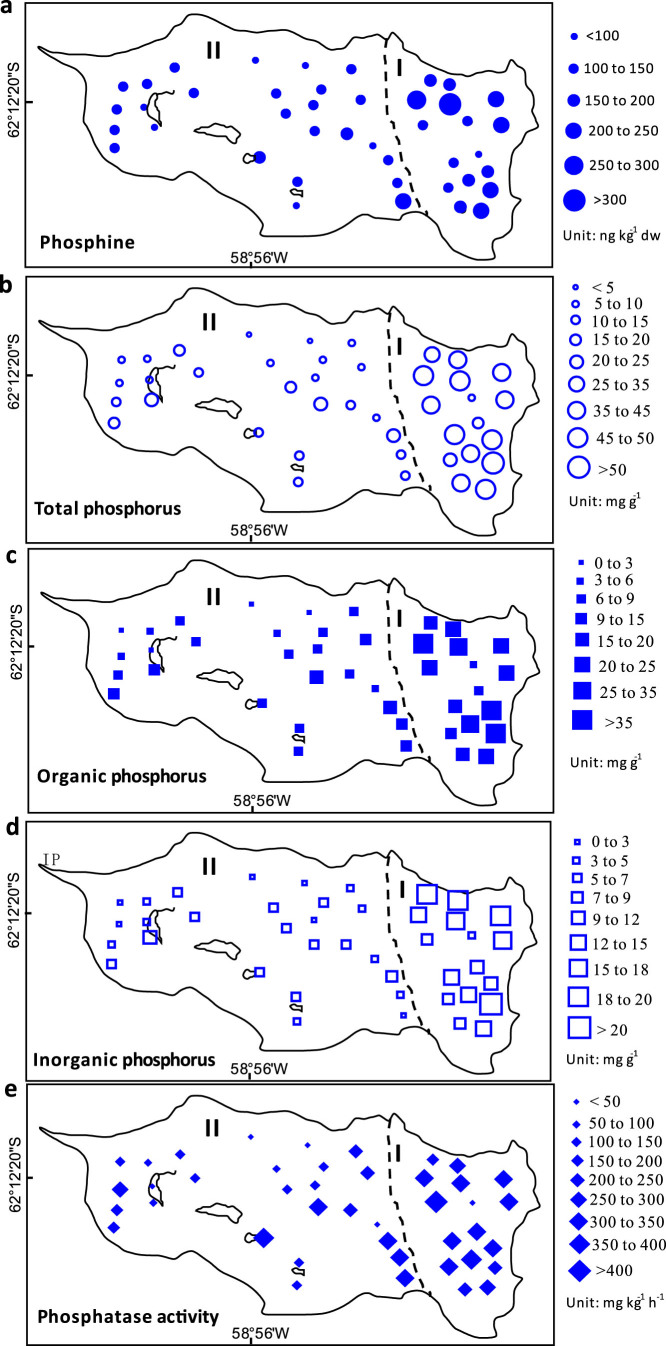
Effects of penguin activities on the spatial distribution patterns for mean concentrations of matrix-bound phosphine (a), phosphorus fractions (b, c, d) and phosphatase activity (e) in tundra soils on Ardley Island. I. Active penguin colony tundra; II. The adjacent tundra lacking visible penguin and other animal activities. The boundary of areas I and II was determined according to our field investigation in Antarctica^34^. *The figures were drawn using Microsoft Excel 2010 and then converted to eps format using Microsoft Office Visio 2007.

**Figure 3:**
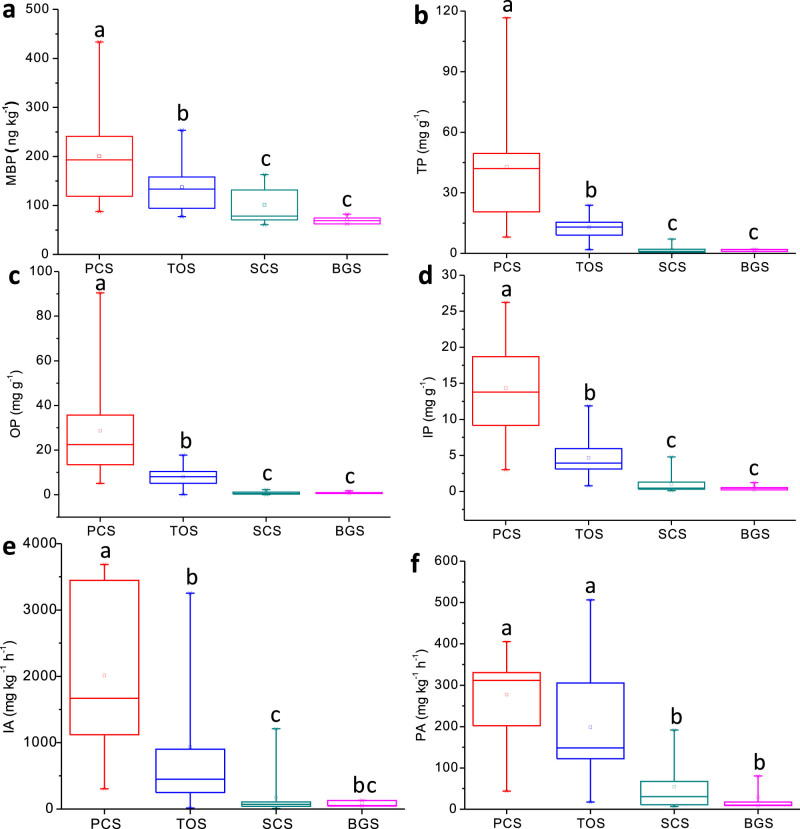
Penguin activities significantly increased tundra soil MBP formation by altering soil phosphorus biogeochemical properties in maritime Antarctica. (a) MBP: matrix-bound phosphine; (b) TP: Total phosphorus; (c) OP: Organic phosphorus; (d) IP: Inorganic phosphorus; (e) IA: Invertase activity; (f) PA: Phosphatase activity. PCS, TOS, SCS and BGS indicated penguin colony soils, penguin-lacking tundra ornithogenic soils, seal colony soils and the background soils. The squares represent means and solid lines represent median values. Box enclose the interquartile range, whiskers show the full range. The different lowercase letters indicate statistically significant differences between means within each type of sites (Fisher's LSD, P<0.05).

The study area was on Ardley Isalnd and Fildes Peninsula (61°51′S-62°15′S, 57°30′W-59°00′W) in the southwest of King George Island, belonging to the so-called maritime Antarctica (Fig. 1). This area is characterized by oceanic climate. According to the meteorological data from 2000 to 2010 at Chinese Great Wall Station located on Fildes Peninsula, mean annual temperature was about −2.5°C with a daily mean range from −26.6 to 11.7°C, and mean annual precipitation was 630 mm mainly in the form of snow. Ardley Island is connected with Fildes Peninsula by a sand dam, with an area of about 2.0 km^2^. It is one of the most important penguin colonies in maritime Antarctica. It is of particular importance for the breeding colonies of Gentoo penguins (*Pygoscelis papua*), Adélie penguins (*Pygoscelis adeliae*) and Chinstrap penguins (*Pygoscelis antarctica*)^34^. Fildes Peninsula is one of important marine animal colonies with an area of about 30 km^2^. According to annual statistical data, a total of over 10,700 marine animals colonized on this peninsula every summer. On the western coast are some established colonies of marine mammals, including five pinnipeds of Weddell seal (*Leptonychotes weddellii*), elephant seal (*Mirounga leonine*), leopard seal (*Hudrurga leptonyx*), fur seal (*Arctocephalus gazella*) and crabeater (*lobodon carcinophagus*)^37^. During the breeding period each summer, penguin guano and seal excreta were deposited into tundra soils or catchment sediments by snow-melt water. Mosses and lichens predominate over the local vegetation. However, the vegetation is highly absent in animal colonies due to the overmanuring and penguin or seal trampling, and only some coprophilic algae grow there^34^.

### Experimental design and soil sample collection

In the summer of 2011/2012, soil samples with a depth of about 10 cm were collected from the active penguin colony (n = 16) and its adjacent tundra lacking penguin and other animal activities (n = 26) on Ardley Island, the active seal colony and its adjacent tundra lacking seal and other animal activities (n = 18) along the western coast of Fildes Peninsula using a clean bamboo scoop. The adjacent tundra areas are located outside the penguin or seal colony but where penguins, seals or other animals occasionally wander. The background tundra soils (n = 4) were sampled from upland tundra and the preglacial area on Nelson Island far away from marine animal colonies as the control to study effects of penguin and seal activities on soil phosphine production and emissions. These soils had not been disturbed and kept intact. Immediately after collection, all samples were sealed in the clean plastic bags and stored in the dark at −20°C until laboratory analysis. The soil samples were divided into two portions. One portion was used to analyze soil MBP concentrations and PH_3_ production rates, whereas the other portion was used to determine phosphorus fractions, enzyme activities, bacterial abundance and other soil physicochemical properties. The samples were measured within one month after the soils were operated.

### Determination of matrix-bound phosphine

Briefly, about one gram of wet soils was digested in a glass reactor with 5 ml of 0.5 mol L^−1^ H_2_SO_4_ for 5 min at 100°C under N_2_. The liberated phosphine was purged with pure N_2_ out of the reactor into a 30 ml syringe and transferred into the gas chromatograph (Varian CP 3800) after enrichment through a capillary cryo-trapping (a Plot-Q capillary column, cooled down with liquid nitrogen)^14^,^19^. The gas chromatograph was equipped with a capillary 121 column (crosslinked 5% Ph Me Silicone, 25 m × 0.2 mm × 0.33 μm film thickness, Hewlett Packard) and a pulsed flame photometric detector (PFPD). The method has a detection limit of 0.1 ng m^−3^ of phosphine. Each gas sample was measured at least three times with a relative standard deviation of less than 10%. The mass of phosphine in this gas divided by the mass of dry soil sample yields MBP.

MBP in soil is commonly assumed to include absorbed phosphine, metal-phosphine complexes and inorganic phosphides that can be set free as PH_3_ by analytical digestion or through bacterial action^2^,^16^. MBP was analyzed because it is linked to production, consumption and emission of free phosphine in the soils. It is assumed to indicate a stationary state concentration of phosphine between production and consumption under natural conditions^2^.

### Field phosphine flux measurement

**Figure 4:**
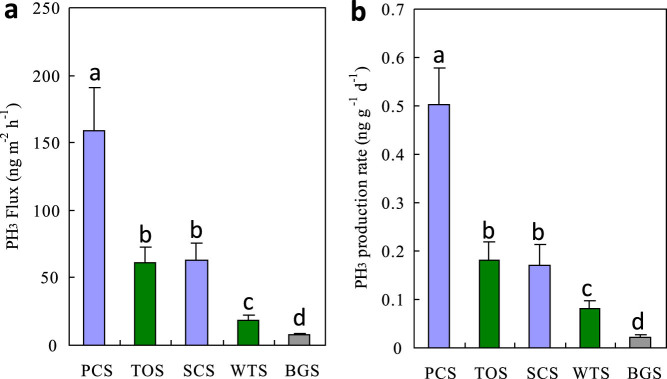
Phosphine fluxes (a) and its production rate (b) from penguin colony soils (PCS), the adjacent penguin-lacking tundra soils (TOS), seal colony soils (SCS), the adjacent seal-lacking tundra soils (WTS) and the background soils (BGS). (a) In-situ phosphine fluxes (n = 6); (b) In-vivo phosphine production rate from the soils at 4°C (three repetitions).

**Figure 5:**
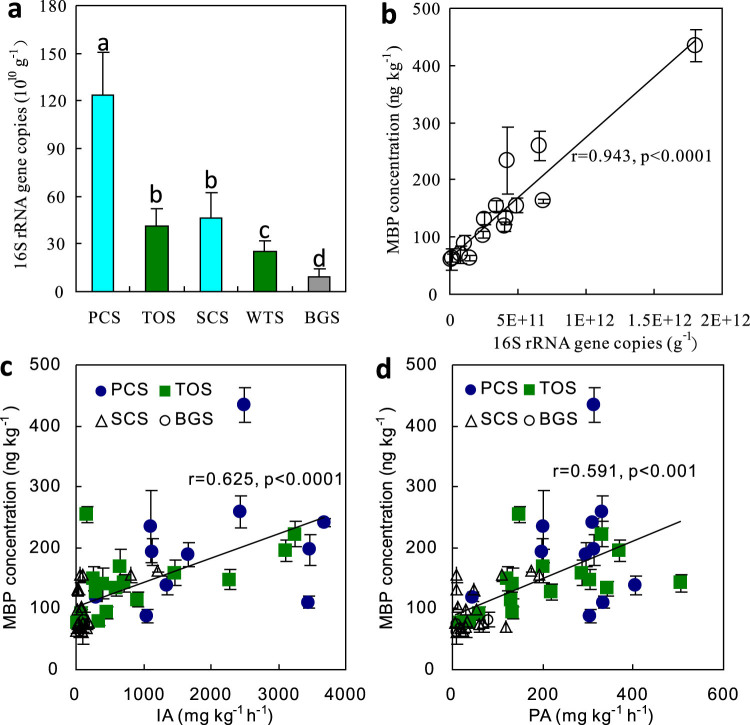
Correlation between MBP levels and bacterial abundance, enzyme activities in tundra soils of maritime Antarctica. Note: r and p present Spearman's rank correlation coefficient and the significant level between the correlations of MBP and the parameters, respectively. PCS, TOS, SCS, WTS and BGS indicated penguin colony soils, the adjacent penguin-lacking tundra soils, seal colony soils, the adjacent seal-lacking tundra soils and the background soils, respectively.

During the summer of 2011/2012, two observation sites for PH_3_ fluxes were established in each type of tundra areas, respectively, including active penguin colony and its adjacent tundra lacking penguin and other animal activities, active seal colony and its adjacent tundra lacking seal and other animal activities, and the background tundra (as the control) far away from marine animal colonies. The closed-chamber method^6^,^14^,^30^ was used for the measurement of soil phosphine fluxes from each type of sites above. Chambers were made of opaque plastics to avoid phosphine photo-degradation. During flux measurements, the chambers were put on the bottom collars inserted into the soil with a cross-sectional area of 0.25 m^2^ (50 × 50 cm). The internal height of the chamber is about 20 cm. Two parallel chambers were employed for flux measurements at each observation site (Two repetitions), and their mean flux was used as the result. A gas sample was taken with 50-ml disposable polypropylene-syringe immediately after the chamber was put on the collar, and then continuously transferred into a Tedlar bag through a three-port valve. A second and third gas sample was taken usually 20, 40 min after the first. Due to lower Antarctic temperature, 40 min was used for phosphine flux measurements, which had an insignificant effect on the microclimate in the chamber according to our previous studies^6^. The fluxes were measured three times at each type of sites (n = 2) on December 31, 2011, January 14 and January 26, 2012. Total six fluxes were obtained for each type of sites, and their mean fluxes were used to compare the difference between the site types (Fig. 4). The fluxes were calculated from the temporal increase of phosphine concentration inside the chambers according to the following equation^6^,^30^: 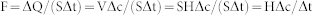
where F is the flux or the emission rate in nanograms per square meter per hour; c is the PH_3_ mass concentration in nanograms per cubic meter; t is the sampling duration in hours; H, S and V are, respectively, the effective height, effective cross-sectional area and effective volume of a chamber in meters, square meters and cubic meters; and ΔQ is the difference of PH_3_ quantity existing in the chamber with time in nanograms.

### Laboratory simulation of phosphine production

The soils (about 100 g) were added into 100 mL glass bottles, both filled with high-purity nitrogen and sealed anaerobically. The bottles were incubated statically in a thermostatic chamber in the dark for 72 h; the temperatures were kept at 4°C. The headspace gas samples were taken four times every day for analyzing gaseous phosphine. The simulation conditions (4°C, dark, anaerobic) are according to tundra soils in the summer. Tundra soil temperature is very close to 4°C, and soil moisture is high due to frequent precipitation, which can keep the soils in an anaerobic environment^34^.

### Analyses of soil P fractions and enzyme activity

Phosphorus fractions in the soils were measured as total phosphorus (TP), inorganic phosphorus (IP) and organic phosphorus (OP). TP was analyzed by measuring phosphate using the ammonium molybdate spectrophotometric method after digestion, in which all the samples were dried at room temperature, and then incinerated in a muffle furnace at 550°C for 2 h, followed by the extraction with 1 mol L^−1^ hydrochloric acid for 16–18 h. IP was detected by the same method as TP except the incinerating procedure, and OP was obtained by the difference between TP and IP^6^,^27^.

Soil enzyme activities were based on the release and quantitative determination of the product in the reaction mixture when soil samples were incubated with substrate and buffer solution. The 2.5 g of soils were added into a 25 mL Erlenmeyer flask and then treated with 0.1 mL of toluene, 5 mL of pH = 6 modified universal buffer, and 5 mL of 5% sucrose solution, and the invertase activity was measured using 3, 5-Dinitrosalicylic acid method^33^. The samples (2.5 g) were weighed and put into a 100 mL volumetric flask. The reaction mixture consisted of 2.5 mL of benzene disodium (25 mg mL^−1^) and 2.5 mL borate buffer, and phosphatase activity was determined by the release of p-nitrophenol from p-nitrophenyl phosphate (only neutral phosphatase activity was determined due to pH ranges from 6 to 8)^32^. All the samples were incubated for 24 h at 37°C, then filtered, and thus measured spectrophotometrically at 578 nm, and soil enzyme activity was expressed as mg kg^−1^ h^−1^.

### Real-time quantitative PCR of bacterial 16S rRNA genes

The copy number of soil bacterial genes in penguin colony, seal colony, their adjacent animal-lacking tundra and the background sites was quantified by a BIO-Rad CFX96 real-time PCR system^32^,^35^. PCR mixtures (20 μl) contained 10 μl of SYBR green master mix, 0.5 μM of each primer, 1 μl of DNA template (2 ng μl^−1^), and 6 μl of ultrapure water for balance. The reaction condition had three minutes of initial denaturing step to activate the DNA polymerase at 95°C, followed by 45 cycles of 10 s of denaturing at 95°C, 20 s of primer annealing at 58°C, and 30 s of primer extension at 72°C. Plasmid quantification standards were constructed for the primer pair by cloning PCR products resulting from the amplification of environmental DNA. Exact matches between 16S rRNA sequences of PCR product inserted, and qPCR primer pairs were confirmed by sequencing. DNA copy numbers of PCR product inserted containing in clones were calculated, and standard curves were generated by serial dilution (3.0 × 10^3^ to 3.0 × 10^8^ DNA copies of template per assay). The amplification of the environmental samples and standards, including the controls containing no DNA template (ultrapure water only), was done in triplicate. The average amplification efficiency for Bacteria was 77.3%, and the amplifications were linear (r^2^ = 0.995).

### General analysis of soil other characteristics

The soil samples were mixed homogeneously for the general analyses. The mean grain size (Mz) of the soils was determined by using a laser diffraction particle size analyzer (LS I3 320). Soil gravimetric moisture content (Mc) was determined by drying the soil at 105°C for 12 h, and calculated as: Mc = (mass before drying-mass after drying)/mass after drying × 100%. The pH was determined by ion selection electrode using a soil-to-water ratio of 1:3. Total carbon (TC), total nitrogen (TN) and total sulfur (TS) were determined by using a CNS Elemental Analyzer (Vario EL III) with a relative error of 0.1%. The NH_4_^+^–N and NO_3_^−^–N contents were analyzed using the colorimetric indophenol blue method and Griess–Ilosvay colorimetric method^34^,^36^.

### Statistical analysis

The mean values and standard deviation (mean ± sd) were calculated to facilitate comparisons of the data between the different samples. Compared to the local background soils (BS), the contribution rates (CR) of penguins or seals to soil MBP and P fractions (PS or SS) were calculated as: CR_P_ = (C_PS_-C_BS_)/C_PS_ × 100% for penguins, and CR_S_ = (C_SS_-C_BS_)/C_SS_ × 100% for seals. The C_PS_, C_SS_ and C_BS_ indicate the concentrations of MBP and P fractions in penguin colony soils, seal colony soils and the background soils, respectively. The ratios of phosphine fluxes (R_f_) and production rates (R_pr_) from penguin or seal colony soils to those from the background soils were calculated to indicate effects of penguin or seal on soil PH_3_ fluxes and production rates. Correlation coefficients between MBP concentrations and phosphorus fractions, enzyme activities, and other environmental factors were analyzed using Person correlation. The factors tested and the relationship were considered statistically significant where *P*<0.05. Differences in mean concentrations of MBP and environmental parameters between the soils of different tundra areas were tested with Student's *t*-test at *P* = 0.05. All statistical analyses were preformed using Microsoft Excel 2007, OriginPro 8.0 and SPSS 16.0 for Window XP.

## Author Contributions

R.B.Z., Q.W. and W.D. prepared the primary manuscript, figures and simulation experiments. W.D., Q.W. and C.W. analyzed the phosphorus factions and soil characteristics. D.W.M. collected the samples from the Antarctica and analyzed soil bacterial abundance. L.J.H. measured the phosphine concentrations in the samples. All authors reviewed and discussed the manuscript.

## Supplementary Material

Supplementary Information Supplementary Material

## Figures and Tables

**Table 1:** Contribution rates of penguin and seal to soil matrix-bound phosphine and phosphorus fractions compared to the background Antarctic soils

Soil types	Penguin or seal contribution rates (%)			
	TP	OP	IP	MBP
Penguin colony soils	95.8	98.0	95.3	62.3
Adjacent penguin-lacking tundra soils	88.2	93.0	85.7	45.1
Seal colony soils	74.7	72.4	76.5	25.7
Adjacent seal-lacking tundra soils	34.8	41.7	27.3	12.8

**Table 2:** Ratios of phosphine fluxes and production rates from penguin or seal colony soils to those from the local background Antarctic soils. Note: R_f_, the ratio of PH_3_ fluxes from penguin or seal colony soils to those from local background soils; R_pr_, the ratio of PH_3_ production rates

Soil types	R_f_	R_pr_
Penguin colony soils	21.3	25.1
Adjacent penguin-lacking tundra soils	8.2	9.0
Seal colony soils	8.4	8.6
Adjacent seal-lacking tundra soils	2.5	4.0
