# Supplementary material for: Penguins significantly increased phosphine formation and phosphorus contribution in maritime Antarctic soils
Source: Sci Rep. 2014 Nov 14;4:7055. doi: 10.1038/srep07055 (PMC4231338; doi:10.1038/srep07055)
Supplement: Supplementary Information — Supplementary Material [file srep07055-s1.doc]

**Supplementary Material**

**Penguins significantly increased phosphine formation and phosphorus contribution in maritime Antarctic soils**

Renbin Zhu^1^,*, Qing Wang^1^, Wei Ding^1^, Can Wang^1^, Lijun Hou^2^ & Dawei Ma^1^

^1^Institute of Polar Environment, School of Earth and Space Sciences, University of Science and Technology of China, Hefei City, Anhui Province 230026, P. R China,

^2^State Key Laboratory of Estuarine and Coastal Research, East China Normal University, Shanghai 200062, P. R China

*Corresponding author: Email: zhurb@ustc.edu.cn; Tel: 0086-551-63606010; Fax: 0086-551-63606010.

**Figure S1** **Relationships between MBP levels and environmental vaiables in tundra soils.** Note: r and p present Spearman’s rank correlation coefficient and the significant level between the correlations of MBP and other parameters, respectively. Note: MBP: matrix-bound phosphine; TP: total phosphorus; OP: organic phosphorus; IP: inorganic phosphorus; TN: total nitrogen; TOC: total organic carbon; TS: total sulfur. PCS, TOS, SCS and BGS indicated penguin colony soils, penguin-lacking tundra ornithogenic soils, seal colony soils and the background soils.

**Table S1 Physicochemical characteristics of tundra soils in the different tundra areas of maritime Antarctica**

| Sampling No. | Mz (µm) | pH | TC (%) | TN (%) | TS (%) | NH_4_^+^ (μg g^-1^) | NO_3_^-^ (μg g^-1^) | C/N |
| --- | --- | --- | --- | --- | --- | --- | --- | --- |
| Penguin colony soils | | | | | | | | |
| PS1 | - | 6.1 | 11.4 | 1.75 | 0.19 | 1532.36 | 0.91 | 6.5 |
| PS3 | 135.91 | 5.4 | 9.0 | 1.10 | 0.17 | 201.77 | 1.12 | 8.2 |
| PS4 | - | - | 25.9 | 4.31 | 0.61 | 1216.23 | 3.37 | 6.0 |
| PS6 | - | - | 19.6 | 3.24 | 0.66 | 2793.67 | 4.69 | 6.0 |
| PS8 | - | 5.9 | 17.8 | 2.83 | 0.48 | 1596.04 | 4.99 | 6.3 |
| PS10 | - | - | 31.3 | 4.39 | 0.55 | - | - | 7.1 |
| PS12 | - | 5.9 | 26.7 | 3.79 | 0.56 | 223.99 | 2.34 | 7.1 |
| PS13 | 97.896 | - | 14.9 | 1.67 | 0.22 | 233.27 | 1.42 | 8.9 |
| PS14 | - | 5.9 | 17.5 | 2.25 | 0.32 | 2256.18 | 8.53 | 7.8 |
| PS16 | 100.71 | 5.4 | 3.8 | 0.40 | 0.16 | 149.51 | 1.14 | 9.3 |
| PS18 | 95.91 | 6.2 | 17.8 | 2.87 | 0.54 | - | 0.91 | 6.2 |
| The adjacent penguin-lacking tundra soils | | | | | | | | |
| TS1 | 71.8 | 5.3 | 19.5 | 1.66 | 0.30 | 72.59 | 2.47 | 11.8 |
| TS2 | 77.8 | - | 17.3 | 1.61 | 0.28 | 65.88 | 2.11 | 10.8 |
| TS3 | 41.6 | 5.5 | 17.4 | 1.57 | 0.24 | 56.42 | 2.47 | 11.1 |
| TS4 | 179.2 | - | 3.2 | 0.35 | 0.08 | 8.20 | 1.35 | 9.3 |
| TS5 | 49.8 | 5.8 | 15.9 | 1.47 | 0.22 | 62.28 | 1.24 | 10.8 |
| TS7 | 30.5 | 5.2 | 5.3 | 0.60 | 0.09 | 154.36 | 2.32 | 8.8 |
| TS9 | 49.3 | 5.8 | 8.4 | 0.87 | 0.13 | 1538.66 | 1.51 | 9.6 |
| TS11 | 114.8 | 8.3 | 1.0 | 0.01 | 0.03 | 14.26 | 7.11 | 91.9 |
| TS13 | 126.9 | - | 9.8 | 0.99 | 0.12 | 33.34 | 2.30 | 9.9 |
| TS15 | 190.0 | 5.5 | 7.6 | 0.81 | 0.23 | 2.60 | 2.03 | 9.4 |
| TS16 | 104.3 | 5.3 | 6.7 | 0.70 | 0.15 | 2.43 | 2.88 | 9.6 |
| TS17 | 81.34 | 5.5 | 10.9 | 1.46 | 0.30 | 56.22 | 1.93 | 7.5 |
| TS19 | 148.9 | - | 10.1 | 0.99 | 0.14 | 26.15 | 1.19 | 10.2 |
| TS21 |  | 6.1 | 2.9 | 0.34 | 0.05 | 94.41 | 1.05 | 8.6 |
| TS23 |  | - | 22.3 | 1.48 | 0.40 | 63.37 | 2.89 | 15.0 |
| TS24 | 149.9 | 5.9 | 4.6 | 0.41 | 0.08 | 23.73 | 32.51 | 11.1 |
| TS25 | 148.2 | 6.6 | 0.7 | 0.15 | 0.07 | 8.17 | 4.06 | 5.1 |
| TS26 | 90.9 | 5.8 | 2.4 | 0.34 | 0.12 | 7.79 | 5.75 | 7.0 |
| Seal colony and adjacent tundra soils | | | | | | | | |
| SS1 | 63.5 | - | 1.1 | 0.13 | 0.06 | 21.77 | 0.94 | 8.4 |
| SS2 | 227.7 | - | 0.1 | 0.003 | 0.002 | 0.78 | 1.25 | 21.8 |
| SS3 | 136.4 | - | 0.2 | 0.03 | 0.005 | 4.73 | 0.87 | 5.6 |
| SS4 | 220.5 | 6.7 | 1.9 | 0.41 | 0.15 | 656.41 | 3.34 | 4.6 |
| SS5 | 352.3 | 6.5 | 3.2 | 0.72 | 0.33 | 569.25 | 0.77 | 4.5 |
| SS6 | 234.1 | 6.6 | 2.2 | 0.46 | 0.26 | 162.99 | 0.61 | 4.8 |
| SS7 | 225.9 | 6.4 | 1.6 | 0.34 | 0.18 | 804.49 | 0.95 | 4.7 |
| SS8 | 106.1 | 7.3 | 1.5 | 0.34 | 0.12 | 48.96 | 1.51 | 4.4 |
| SS9 | 115.5 | 7.8 | 4.9 | 1.05 | 0.16 | 21.91 | 1.89 | 4.7 |
| SS10 | - | 7.5 | 9.0 | 1.81 | 0.32 |  | - | 5.0 |
| SS11 | 370.8 | 7.1 | 0.2 | 0.04 | 0.01 | 5.44 | 5.67 | 6.6 |
| SS12 | 222.4 | 7.2 | 0.6 | 0.11 | 0.02 | 81.99 | 0.68 | 5.4 |
| SS13 | 328.2 | - | 1.1 | 0.31 | 0.24 | 731.53 | 1.96 | 3.6 |
| SS14 | 145.4 | - | 0.3 | 0.07 | 0.03 | 353.02 | 2.76 | 4.1 |
| SS15 | 240.7 | - | 0.2 | 0.05 | 0.03 | 9.22 | 3.31 | 4.0 |
| SS16 | 268.9 | 7.5 | 0.1 | 0.03 | 0.02 | 61.82 | 7.68 | 3.9 |
| SS17 | 276.3 | - | 0.1 | 0.02 | 0.02 | 6.19 | 7.55 | 4.0 |
| SS18 | 355.7 | - | 0.1 | 0.03 | 0.01 | -0.17 | 4.88 | 2.9 |
| The background tundra soils | | | | | | | | |
| BS1 | 215.5 | 6.4 | 0.8 | 0.09 | 0.04 | 3.07 | 1.16 | 8.9 |
| BS2 | 403.7 | 7.2 | 0.5 | 0.07 | 0.02 | 8.31 | 2.79 | 7.2 |
| BS3 | 394.1 | 6.9 | 0.1 | 0.04 | 0.02 | 2.41 | 0.83 | 2.7 |
| BS4 | 211.1 | 7.1 | 0.5 | 0.06 | 0.01 | 0.52 | 2.72 | 8.7 |

**Table S2** Correlations between physicochemical characteristics in the soils

| Variables | Mz | Mc | pH | TN | TC | S | NH_4_^+^ | NO_3_^-^ | TP | IP | OP | IA | PA |
| --- | --- | --- | --- | --- | --- | --- | --- | --- | --- | --- | --- | --- | --- |
| Mz | 1 | -0.58** | 0.4** | -0.51** | -0.56** | -0.22 | 0.05 | 0.41** | -0.5** | -0.55** | -0.63** | -0.47** | -0.54** |
| Mc |  | 1 | -0.59** | 0.86** | 0.91** | 0.84** | 0.44** | -0.39** | 0.69** | 0.7** | 0.66** | 0.79** | 0.76** |
| pH |  |  | 1 | -0.34* | -0.5** | -0.32* | 0.05 | 0.58** | -0.51** | -0.40* | -0.53** | -0.41* | -0.58** |
| N |  |  |  | 1 | 0.94** | 0.92** | 0.61** | -0.33* | 0.79** | 0.83** | 0.74** | 0.81** | 0.72** |
| C |  |  |  |  | 1 | 0.86** | 0.42** | -0.37** | 0.71** | 0.74** | 0.66** | 0.82** | 0.81** |
| S |  |  |  |  |  | 1 | 0.65** | -0.37** | 0.77** | 0.80** | 0.73** | 0.67** | 0.63** |
| NH_4_^+^ |  |  |  |  |  |  | 1 | -0.25* | 0.52** | 0.55** | 0.52** | 0.38** | 0.33* |
| NO_3_^-^ |  |  |  |  |  |  |  | 1 | -0.24 | -0.22 | -0.22 | -0.30 | -0.45 |
| TP |  |  |  |  |  |  |  |  | 1 | 0.92** | 0.99** | 0.53** | 0.54** |
| IP |  |  |  |  |  |  |  |  |  | 1 | 0.84** | 0.56** | 0.61** |
| OP |  |  |  |  |  |  |  |  |  |  | 1 | 0.49** | 0.52** |
| IA |  |  |  |  |  |  |  |  |  |  |  | 1 | 0.76** |
| PA |  |  |  |  |  |  |  |  |  |  |  |  | 1 |

**Table S3** An overview of MBP concentrations in soils and aquatic sediments

| Sample type | Location | MBP concentration (ng kg^-1^) | | References |
| --- | --- | --- | --- | --- |
|  |  | Range | Average |  |
| *Soil* | | | | |
| Penguin colony soils | Ardley Island, west Antarctica | 87.4–433.6 | 200.3 | This study |
| Adjacent penguin-lacking soils | Tundra on Ardley Island, west Antarctica | 78.4-253.4 | 137.4 | This study |
| Seal colony soils | Fildes Peninsula, west Antarctica | 63.2–156.2 | 101.6 | This study |
| Background soils | Fildes Peninsula, west Antarctica | 62.4 –89.8 | 75.5 | This study |
| Soils, industrial area | German | 18.4-103.3 |  | Eismann et al. (1997)^1^ |
| Soils, Rural area | German | 0.8-17.1 |  | Eismann et al. (1997)^1^ |
| Paddy soil | Beijing, China | 10.4-44.7 |  | Han et al. (2000)^2^ |
| Paddy soil | Jiangsu Province, China | 17.0-1035 | 189±44 | Zhang et al. (2010)^3^ |
| Ornithogenic soil | West Antarctica | 0.5-13.34 |  | Zhu et al. (2006)^4^ |
| Soil of virgin tropical forest | Mahé, Seychelles | 1-5 |  | Glindemann et al. (2005)^5^ |
| *Freshwater sediments* | | | | |
| Freshwater river surface Sediments | Hamburg harbour, Germany | 0.2-56.1 |  | Gassmann and Schorn (1993)^6^ |
| Freshwater river surface Sediments | Hamburg harbour, Germany | 43-201 | 99 | Gassmann (1994)^7^ |
| Freshwater river subsurface sediments | Hamburg harbour, German | 93-753 | 216 | Gassmann (1994)^7^ |
| Freshwater river sediment | Elster river, Germany | 4-1140 | 227 | Glindemann et al. (2005)^5^ |
| Freshwater lake sediment | Tanhu, China | 5.4-919.2 | 185 | Geng et al. (2005)^8^ |
| Ornithogenic lake sediments | Y2 lake in Ardley Island, western Antarctica | 0.29-3.04 |  | Zhu et al. (2006)^4^ |
|  |  |  |  |  |
| *Marine sediments* | | | | |
| Marine surface sediments | German bright, North Sea, German | 158-1923 | 770 | Gassmann (1994)^7^ |
| Marine subsurface sediments | German bright, North Sea, German | 81-2217 | 853 | Gassmann (1994)^7^ |
| Marine surface sediments | Coastal areas, Jiaozhou Bay, China | 125-591 | 290 | Yu and Song (2003)^9^ |
| Marine subsurface sediments | Coastal areas, Jiaozhou Bay, China | 192-545 | 400 | Yu and Song (2003)^9^ |
| Estuarine subtidal sediments | Yangtze Estuary, China | 2-95 | 17 | Feng et al. (2008a)^10^ |
| Marine surface sediments | Marine areas along Chinese coast | 0.89-25.86 |  | Feng et al. (2008b)^11^ |
| Estuarine intertidal sediments | Yangtze Estuary, China | 0.65-3.25 | 1.53 | Hou et al. (2009)^12^ |
| Coastal surface sediments | Southwest Yellow Sea | 0.69-179 |  | Hong et al. (2010)^13^ |
| Surface sediment | Yellow Sea | 0.19-38.24 | 4.2 | Li et al. (2010b)^14^ |
| Surface sediment | Changjiang Estuary and its adjacent shelf area | 0.1-29.7 | 10.9 | Li et al. (2010a)^15^ |

**References**

1. Eismann, F., Glindemann, D., Bergmann, A. & Kuschk, P. Soils as source and sink of phosphine. *Chemosphere* **35**, 523–533 (1997).

2. Han, S. H., Zhuang, Y. H., Liu, J. A., Glindemann, D. Phosphorus cycling through phosphine in paddy fields. *Sci. Total Environ.* 258: 195–203 (2000).

3. Zhang, J., Geng, J. J., Zhang, R., Ren, H. Q. & Wang, X. R. Matrix-bound phosphine in paddy fields under a simulated increase in global atmospheric CO_2_. *Environ. Chem.* **7**, 287–291 (2010).

4. Zhu, R. B. *et al.* Matrix-bound phosphine in Antarctic biosphere. *Chemosphere* **64**, 1429–1435 (2006).

5. Glindemann, D., Edwards, M., Liu, J. & Kuschk, P. Phosphine in soils, sludges, biogases and atmospheric implications–a review. *Ecol. Eng.* **24**, 457–463 (2005).

6. Gassmann, G. & Schorn, F. Phosphine from harbor surface sediments. *Naturwiss* **80**, 78–80 (1993).

7. Gassmann, G. Phosphine in fluvial and marine hydrosphere. *Mar. Chem.* **45**: 197–205 (1994).

8. Geng, J. J. et al. Matrix bound phosphine formation and depletion in eutrophic lake sediment fermentation–simulation of different environmental factors. *Anaerobe* **11**, 273–279 (2005).

9. Yu, Z. M. & Song, X. X. Matrix-bound phosphine: a new form of phosphorus found in sediment of Jiaozhou Bay. *Chin. Sci. Bull.* **48**: 31–35 (2003).

10. Feng, Z., Song, X. & Yu, Z. Seasonal and spatial distribution of matrix-bound phosphine and its relationship with the environment in the Changjiang River estuary. *Mar. Pollut. Bull.* **56**, 1630–1636 (2008a).

11. Feng, Z., Song, X. & Yu, Z. Distribution characteristics of matrix-bound phosphine along the coast of China and possible environmental controls. *Chemosphere* **73**, 519–525 (2008b).

12. Hou, L. J., Yang, Y., Jiang, J. M., Lin, X. & Liu, M. Occurrence of matrix-bound phosphine in intertidal sediments of the Yangtze estuary. *Chemosphere* **76**, 1114–1119 (2009).

13. Hong, Y. N. *et al.* Phosphorus fractions and matrix-bound phosphine in coastal surface sediments of the Southwest Yellow Sea. *J. Hazard. Mater.* **181**, 556–564 (2010).

14. Li, J. B., Zhang, G. L., Zhang, J., Liu, S. M. & Ren, J. L. Matrix-bound phosphine in sediments of the yellow sea and its coastal areas. *Cont. Shelf Res.* **30**, 743–751 (2010a).

15. Li, J. B. *et al.* Matrix bound phosphine in sediments of the Changjiang estuary and its adjacent shelf areas. *Estuar. Coast. Shelf Sci.* **90**, 206–211 (2010b).
